# New ways to make a blood cell

**DOI:** 10.7554/eLife.06877

**Published:** 2015-03-12

**Authors:** Ines Anderl, Dan Hultmark

**Affiliations:** Laboratory of Genetic Immunology, BioMediTech, University of Tampere, Tampere, Finland and the Department of Molecular Biology, Umeå University, Umeå, Sweden; Department of Molecular Biology, Umeå University, Umeå, Sweden and the Laboratory of Genetic Immunology, BioMediTech, University of Tampere, Tampere, Finlanddan.hultmark@ucmp.umu.se

**Keywords:** hematopoiesis, Notch signaling, sessile cluster, crystal cell, *D. melanogaster*

## Abstract

In a niche under the skin in *Drosophila* larvae, blood cells called plasmatocytes can transform into other classes of blood cell.

**Related research article** Leitão AB, Sucena É. 2015. *Drosophila* sessile hemocyte clusters are true hematopoietic tissues that regulate larval blood cell differentiation. *eLife* **4**:e06166. doi: 10.7554/eLife.06166

**Image** A hemocyte cluster provides an environment that allows certain blood cells to change into other types

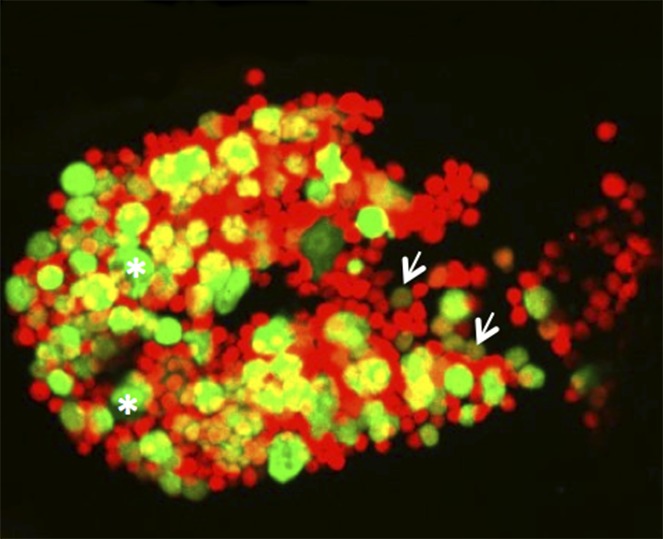

A bewildering variety of different kinds of blood cell are engaged in the immune defense of humans, and they must all be present in the right numbers in the blood. Understanding how blood cells develop in humans and other vertebrates has therefore been a focus of intense research. A picture has emerged in which a small number of self-renewing stem cells give rise to multipotent progenitor cells, from which successive generations of progenitor cells can develop, each progressively more restricted in the types of cell they can develop into. Eventually the progenitor cells give rise to the different classes of mature blood cells (Doulatov et al., 2012; Riether et al., 2015). This one-way hierarchical mode of blood cell formation (or hematopoiesis) takes place in bone marrow and other tissues that provide an environment called a hematopoietic niche. Developmental decisions are controlled by complex interactions between the cells that make up the niche and the developing blood cells.

Similar principles have been shown to operate for hematopoiesis in the model organism *Drosophila melanogaster*. A hematopoietic niche has been identified in the so-called ‘lymph glands’ of *Drosophila* larvae, which are found in an area called the posterior signaling centre. This niche controls the proliferation and specialization—or ‘differentiation’—of adjacent stem cells and/or progenitor cells (Krzemień et al., 2007; Mandal et al., 2007). Now, in *eLife*, Alexandre Leitão and Élio Sucena of the Instituto Gulbenkian de Ciência in Portugal challenge the notion that hematopoiesis occurs through a neat transition from stem cells via progenitor cells to differentiated blood cells. Instead, they show that some differentiated blood cells can transform into blood cells of another class (Leitão and Sucena, 2015).

Three major classes of blood cells, or hemocytes, can be distinguished in *Drosophila*: plasmatocytes, crystal cells and lamellocytes (Figure 1). Most abundant are the plasmatocytes. These are professional phagocytes—cells that engulf and destroy bacteria and cells that are programmed to die. Crystal cells are involved in wound healing and control the production of the black pigment melanin. Finally, lamellocytes are specialized to respond to parasite infection, which they do by forming a capsule around the parasite.

**Figure 1.**
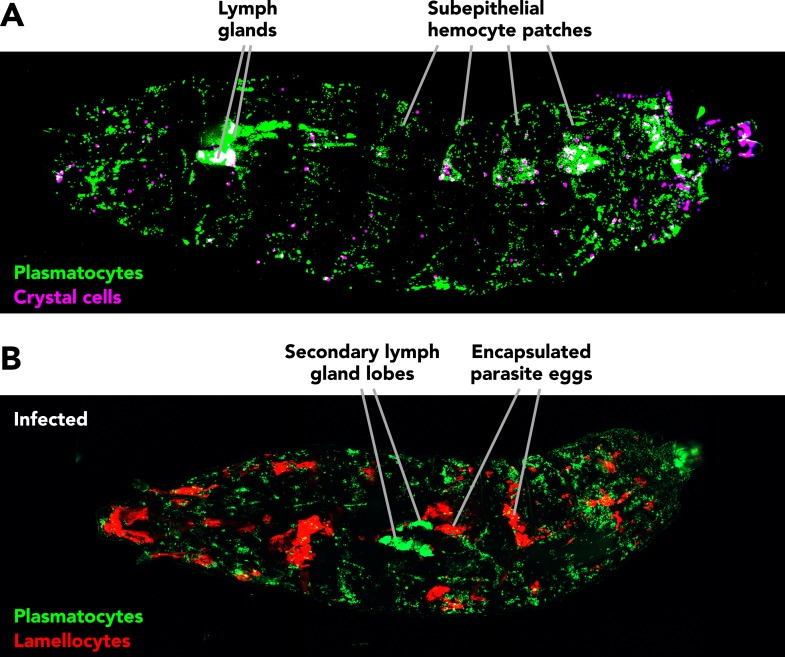
Fluorescent marker genes reveal the different blood cell types in healthy and infected *Drosophila* larvae. (**A**) Blood cells in a healthy larva: plasmatocytes are in green and crystal cells are in magenta; white indicates the presence of both types of cell. Sessile blood cells of the first generation form a pattern of patches under the skin. Second generation blood cells accumulate in the ‘lymph glands’ (which are not related to vertebrate lymph glands in structure or function). Circulating blood cells do not give sharp images. (**B**) Large numbers of lamellocytes (red) accumulate in larvae infected by parasitoid wasp eggs, attaching to the eggs and other parts of the larva. The sessile blood cells become dispersed, the primary lobes of the lymph glands release their contents, and the secondary lobes enlarge.

*Drosophila* hemocytes are generated in two distinct waves during development (Holz et al., 2003). The first generation of hemocytes forms in tissue in the head of the embryo and persists in the fly until adulthood. Embryonic plasmatocytes from this first generation colonize the surfaces of larval tissues, forming sessile clusters of hemocytes that constantly exchange cells with their circulating counterparts. Conveniently, many of these clusters are located in patches under the skin, so researchers can observe them directly (Figure 1A). The plasmatocytes divide and the total number of hemocytes increases gradually during larval development. As shown by Katja Brückner and co-workers in 2011, plasmatocytes proliferate most actively in the sessile subepithelial patches, where cell division appears to be controlled by the peripheral nervous system (Makhijani et al., 2011).

A second generation of hemocytes forms in the lymph glands. Under normal conditions, the lymph glands do not release hemocytes until metamorphosis, at the end of the larval stage. Up to that point all circulating larval hemocytes belong to the first generation of blood cells.

In 1957, Tahir Rizki proposed a model in which parasite infection causes plasmatocytes to transform into lamellocytes; the model also assumes that plasmatocytes and crystal cells independently originate from progenitor cells dubbed prohemocytes (Rizki, 1957). However, work on hematopoiesis in the lymph glands suggested a different model, where all three hemocyte classes originate directly from undifferentiated precursor cells (Evans et al., 2003). Furthermore, the lymph glands respond vigorously to parasite infection, producing lamellocytes and other hemocytes that are prematurely released into circulation (Figure 1B). It was therefore generally assumed that the lymph glands are the major source of cells for the immune defense. However, transplantation and lineage tracing studies have recently shown that most lamellocytes stem directly from first generation larval plasmatocytes (Márkus et al., 2009; Honti et al., 2010; Stofanko et al., 2010), in line with Rizki's original proposal.

Leitão and Sucena have now carefully studied crystal cell development in the subepithelial patches. An obstacle to similar studies in the past has been that larvae are very motile, and their busy peristaltic movements make it difficult to trace the fate of individual cells. Leitão and Sucena solved this problem by using double-sided tape to hold the larvae still. By inserting fluorescent genes into the larvae that produce different colors in different cell types, the developmental fate of individual hemocytes could then be followed for up to three hours using time-lapse imaging. Unexpectedly, Leitão and Sucena discovered that plasmatocytes occasionally transform into crystal cells, at a rate consistent with the increase in the overall number of crystal cells in the larva. Thus, strikingly, hematopoiesis occurs in the subepithelial patches of *Drosophila* larvae without the involvement of stem cells.

A hematopoietic niche is nevertheless required, even in this novel mode of hematopoiesis. Leitão and Sucena show that the decision to begin developing a plasmatocyte into a crystal cell is triggered by signals from neighboring plasmatocytes in the subepithelial patches via the Notch signalling pathway. In a pleasing agreement with this model, the probability that a cell becomes a crystal cell is proportional to the number of plasmatocytes it is in contact with. Furthermore, crystal cell formation is inhibited when hemocytes are physically prevented from settling.

Hence, two distinct types of hematopoiesis coexist in *Drosophila* larvae; one in the lymph glands, where a niche controls the differentiation of stem cells, and another under the skin, where a niche controls how already differentiated plasmatocytes transform into crystal cells and, when needed, lamellocytes.
